# Effectiveness and safety of PD-1/L1 inhibitors as first-line therapy for patients with advanced or metastatic urothelial carcinoma who are ineligible for platinum-based chemotherapy: a meta-analysis

**DOI:** 10.3389/fimmu.2025.1430673

**Published:** 2025-02-12

**Authors:** Weiming Liang, Zhijing Wang, Zhilong Huang, Yanping Huang, Chunyan Li, Yiwen Liang, Miaoyan Huang, Duo Zhang, Chenchen Li

**Affiliations:** ^1^ Department of Medical Oncology, The Sixth Affiliated Hospital, Sun Yat-sen University, Guangzhou, Guangdong, China; ^2^ The First Affiliated Hospital of Guangxi University of Science and Technology, Guangxi University of Science and Technology, Liuzhou, Guangxi, China; ^3^ Department of Urology, Pu'er People's Hospital, Pu'er, Yunnan, China; ^4^ Medicine Center, Guangxi University of Science and Technology, Liuzhou, Guangxi, China

**Keywords:** pembrolizumab, enfortumab vedotin, immunotherapy, urothelial carcinoma, metastatic, objective response rate, meta-analysis, PD-1 inhibitor

## Abstract

**Objective:**

To evaluate the efficacy and safety of programmed cell death protein 1 or its ligand (PD-1/L1) inhibitors as first-line therapy in advanced or metastatic urothelial carcinoma (mUC) who are ineligible for platinum-based chemotherapy.

**Method:**

A systematic search was conducted in four databases (Pubmed, Embase, Web of Science, and the Cochrane Library) to find articles that evaluate the effectiveness of first-line PD-1/L1 inhibitors for mUC, from the establishment of the databases to 22 November 2023. Meta-analyses were performed to evaluate the frequencies of progression-free survival (PFS), overall survival (OS), complete response (CR), partial response (PR), stable disease (SD), progressive disease (PD), objective response rate (ORR), disease control rate (DCR), and grade ≥ 3 treatment-related adverse events (trAEs).

**Results:**

Totally six studies were included for meta-analysis. The CR, PR, SD, PD, ORR, DCR, and grade ≥ 3 trAEs rate were 0.06 [95% confidence interval (CI), 0.04 to 0.07], 0.22 (95% CI, 0.16 to 0.30), 0.27 (95% CI, 0.23 to 0.31), 0.31 (95% CI, 0.20 to 0.44), 0.28 (95% CI, 0.21 to 0.37), 0.57 (95% CI, 0.47 to 0.67) and 0.26 (95% CI, 0.14 to 0.40), respectively. The median PFS and OS were 4.5 months and 13.7 months, respectively. Subgroup analysis showed that PD-1/L1 inhibitors monotherapy had an ORR rate of 0.25 (95% CI, 0.21 to 0.29) and a DCR rate of 0.50(95% CI, 0.44 to 0.56), while PD-1/L1 dual immunotherapy had a better ORR rate of 0.33 (95% CI, 0.15 to 0.52) and a DCR rate of 0.65 (95% CI, 0.49 to 0.80). However, there was no significant difference in PFS and OS between the two groups.

**Conclusion:**

The findings indicated that PD-1/L1 inhibitors could be used as a safe and viable first-line treatment option for patients with advanced or metastatic urothelial carcinoma who were not suitable candidates for platinum-based chemotherapy. Specifically, the combination of Enfortumab vedotin (EV) and pembrolizumab (Pembro) showed more effectiveness in treating patients compared to trials using the current standard treatment, suggesting that it could be a promising alternative treatment option.

**Systematic review registration:**

https://www.crd.york.ac.uk/prospero/, identifier CRD42024510152.

## 1 Introduction

Urothelial carcinoma is on increasing prevalence globally, with a mUC rate of 2.8/100,000 in the US. Unfortunately, patients with advanced or metastatic urothelial carcinoma have a particularly poor prognosis (1–4). Just 5% of patients with mUC manage to survive for five years (5). For all patients who are eligible for cisplatin or carboplatin therapy, platinum-based chemotherapy is the recommended first-line treatment option (6, 7). Platinum-based chemotherapy as a first-line treatment has been shown to enhance the survival rate of patients with mUC (8–10). This approach is widely acknowledged as the most effective first-line treatment (6, 11, 12). Nevertheless, a majority of patients, exceeding 50%, are unable to receive cisplatin due to renal insufficiency, suboptimal physical condition, or other concurrent medical conditions such as hearing loss, neuropathy, or heart failure (11–17).

Furthermore, a significant number of adverse events have been documented in patients who were not eligible for cisplatin treatment and were undergoing platinum-based chemotherapy (14, 15, 18, 19). Gemcitabine plus carboplatin is the predominant treatment combination for patients who are unfit for cisplatin. However, this regimen has demonstrated reduced effectiveness and decreased tolerance, while patients who do not have disease progression are subsequently treated with avelumab (9, 10, 20, 21). Approximately 50% of patients with mUC may not receive any systematic treatment, as indicated by empirical data (22), and most patients with mUC do not undergo chemotherapy because they are primarily concerned about the unpleasant side effects. This emphasizes the necessity for a first-line treatment that is both effective and well-tolerated, and can be administered to a large number of patients in this community (14, 19).

Recently, the utilization of antibodies that target PD-1/L1 has significantly improved the range of treatment options available for the management of mUC (23). PD-1, together with its ligands PD-L1 and PD-L2, are present in different types of solid tumors. These interactions hinder the activity of effector T cells and enable the immune system to evade detection (23–25). Therapeutic drugs that specifically target the PD-1 pathway have shown effectiveness in treating recurring mUC (26–29). In the US, atezolizumab and pembrolizumab are the first-line treatment options advised for individuals who are unable to receive platinum-based chemotherapy (6, 30–34). Both platinum-resistant and untreated mUC were responsive to the anti-PD-L1 antibody durvalumab (35, 36). Nivolumab, a drug that inhibits the PD-1 protein, and Atezolizumab, a drug that inhibits the PD-L1 protein, have demonstrated effectiveness in treating advanced urothelial malignancies (26, 37).

While PD-1/L1 inhibitors may initially provide a long-lasting response in both initial and subsequent treatment stages, the majority of patients with mUC will ultimately experience disease progression and have a generally unfavorable prognosis (38). Combining medicines that target various areas of tumor biology has the potential to overcome drug resistance and enhance the effectiveness of anti-tumor treatment (39). CV301 demonstrated a satisfactory safety profile in Phase I clinical trials, whether used alone or in conjunction with a PD-1 inhibitor (40–42). Preclinical evidence indicated that various antibody-drug conjugates (ADCs), including enfortumab vedotin (EV), when used together with PD-1/L1 inhibitors like Pembro, could increase the effectiveness of anti-tumor activity compared to their individual methods of action, and provide additional support for their combined efficacy (43–45). Lenvatinib, a potent inhibitor of many kinases including VEGF receptor, FGFR receptor, and other receptors, as well as oncogenes, has demonstrated significant antitumor efficacy in solid tumors when administered in conjunction with Pembro (46).

In this study, we conducted a systematic review and meta-analysis to evaluate the efficacy and safety of PD-1/L1 inhibitors as first-line treatment for patients with advanced or metastatic urothelial carcinoma who is not suitable for platinum-based chemotherapy. In addition, since there was still a lack of studies comparing PD-1/L1 inhibitors monotherapy versus PD-1/L1 dual immunotherapy (47), a subgroup analysis was conducted to examine the disparity between PD-1/L1 inhibitors monotherapy and PD-1/L1 dual immunotherapy.

## 2 Materials and methods

### 2.1 Search strategy

The present meta-analysis followed the 2020 guidelines established by the Preferred Reporting Project for Systematic Review and Meta-Analysis (PRISMA).The study has been registered at PROSPERO with the registration number CRD42024510152. A comprehensive search was performed in four databases, including PubMed, Embase, Web of Science, and the Cochrane Library, to retrieve literature published up until November 22, 2023. The search technique adhered to the PICOS principle and utilized a blend of MeSH terms and unrestricted text phrases. The search approach employed was to combine the terms “PD-1/L1 inhibitor”, “urothelial carcinoma”, and “trial”. **Supplementary Material 1** offered a thorough summary of the search record.

### 2.2 Inclusion and exclusion criteria

Inclusion criteria were as follows: (1) patients diagnosed as metastatic or advanced urothelial carcinoma who are ineligible for platinum-based chemotherapy; (2) at least one cohort of patients were administered PD-1/L1 inhibitors as first-line therapy, with or without other immunotherapy; (3) at least one of the following results were documented: CR, PR, SD, PD, ORR, DCR, ORR, OS, PFS and grade ≥ 3 trAEs; (4)Types of studies: randomized controlled trials, single-arm trials.

The exclusion criteria are as follows: (1) other types of articles, such as case reports, publications, letters, comments, reviews, meta-analyses, editorials, animal studies, protocols, conference, etc; (2) other cancers or diseases; (3) not relevant; (4) not first-line treatment; (5) failed to extract data; (6) duplicate patient cohort; (7) patients eligible for platinum-based chemotherapy.

### 2.3 Selection of studies

The procedure of selecting literatures, which included eliminating duplicate entries, was carried out using EndNote (Version 20; Clarivate Analytics). Two independent reviewers conducted the first search. They removed duplicate data, evaluated the titles and abstracts to determine their relevance, and classified each study as either included or excluded. Discussion was performed on the excluded studies and potential biases they might introduce. A resolution was reached by achieving consensus. In the absence of a consensus among the parties, a third reviewer assumed the position of a mediator.

### 2.4 Data extraction

The data was extracted by two reviewers independently. The retrieved data comprised the following data: (1) Basic information of studies, such as the primary author, publication year, country, study methodology, sample size, and primary outcomes; (2) The basic characteristics of the individuals participating in the study, such as the number of patients, their age, and the type of tumor; (3) Outcomes, including of CR, PR, SD, PD, ORR, DCR, ORR, grade ≥ 3 trAEs, Kaplan-Meier curves for OS, and Kaplan-Meier curves for PFS. The discrepancy was resolved by consulting a third investigator for advice. Among the studies included, four cohorts of patients received PD-1/L1 inhibitors monotherapy, such as Pembro, Avelumab and Durvalumab; another four cohorts of patients received PD-1/L1 dual immunotherapy, such as Pembro+Lenvatinib, Durvalumab+Olaparib, Atezolizumab+CV301, Pembro+EV. Given the potential heterogeneity in treatment protocols, a subgroup analysis was conducted based on the treatment protocols to compare the effectiveness and safety PD-1/L1 inhibitors monotherapy versus PD-1/L1 dual immunotherapy.

### 2.5 Quality assessment

Two impartial reviewers assessed the quality evaluation in the studies that were included. For this analysis, we used the modified Jadad scale to evaluate the quality of randomized controlled trials. The single-arm trials were assessed using methodological indicators from non-randomized studies (MINORS). If there were any contradictions, the disputed findings were resolved by group deliberation.

### 2.6 Statistical analysis

The analyses were performed using Stata 12.0 and R version 4.3.1. The analysis utilized the “meta” package and the IPDformKM program. The GetData Graph Digitizer software was employed to extract data from papers including Kaplan-Meier curves, and the individual data were then reconstructed using the IPDformKM utility. The method devised by Guyot et al. was utilized to rebuild patient-specific information on an individual basis (48). The comparison of continuous variables was performed using the weighted mean difference (WMD) and a 95% CI. The study utilized the relative ratio (RR) with a 95% CI to compare binary variables. The medians and interquartile ranges of continuous data were converted to the mean and standard deviation. The statistical heterogeneity among the studies included in the analysis was evaluated using the Cochrane’s Q test and the I^2^ index. Considering that the research included in the analysis were sourced from the public literature, it was generally more rational to select the random effect model as the initial preference. A p-value below 0.05 was considered to have statistical significance.

## 3 Results

### 3.1 Search results

**Figure 1:**
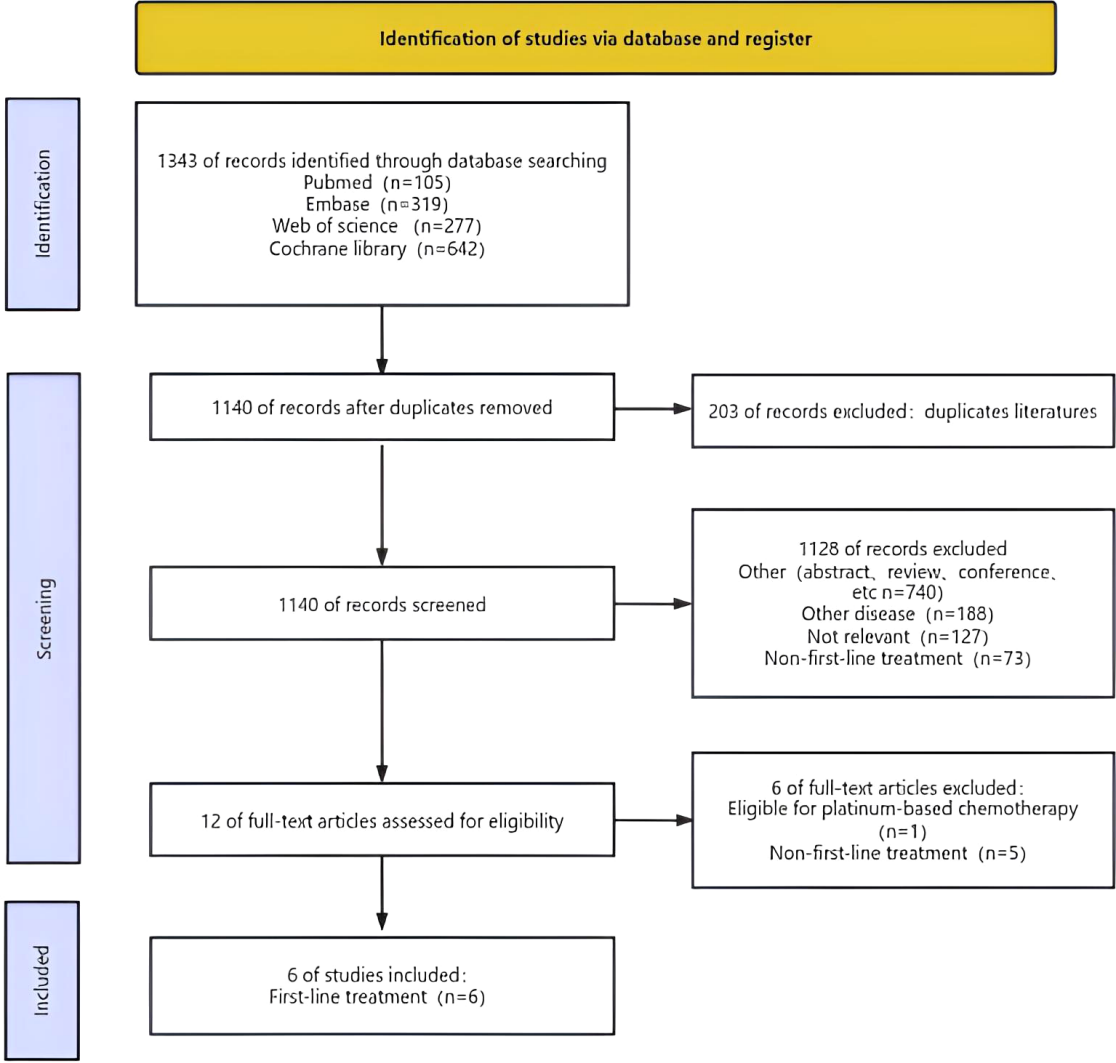
Flow chart of literature search strategies.

**Figure 1** depicted the process of choosing and integrating studies. We initially identified a total of 1,343 studies. Following the removal of redundant research, a total of 1140 articles were retained. Upon evaluating the titles and abstracts, a total of 1128 publications were determined to be irrelevant and thus excluded. After a comprehensive inspection of the entire text, a total of six studies were chosen for inclusion in this meta-analysis.

### 3.2 Patient characteristics and quality assessment

This meta-analysis included a total of six articles (32, 49–53), which consisted of three single-arm trials and three randomized controlled trials. **Table 1** presented detailed data on patient characteristics and quality assessment. The meta-analysis focused exclusively on the data of patients who received PD-1/L1 inhibitors as their first-line treatment. A subgroup analysis was conducted based on the treatment protocols to compare the effectiveness and safety PD-1/L1 inhibitors monotherapy versus PD-1/L1 dual immunotherapy. To be more explicit, the patients were divided into two subgroups according to their individual treatment regimen: subgroup A, patients received PD-1/L1 inhibitors monotherapy; and subgroup B, patients received PD-1/L1 dual immunotherapy. We utilized the modified Jadad scale to assess the quality of RCT literature for quality evaluation purposes. The single-arm studies were assessed using the MINORS tool. All articles were considered to be of high quality.

**Table 1:** Patient characteristics and quality assessment of included studies and patients.

Author, year	Country	Design	Registration ID	Subgroup	Immunotherapy regimen	Cases	Age (Mean ± SD)	Male,%	ECOG PS, n				Quality
									0	1	2	3	
Balar 2017 (32)	USA	single-arm study	NCT02335424	A	Pembrolizumab	370	74 ± 15.00	77.0	80	133	156	1	14
Iacovelli 2022 (51)	Italy	single-arm study	NCT03891238	A	Avelumab	71	NA	77.5	16	33	22	0	13
Matsubara 2023 (52)	Japan	RCT	NCT03898180	A	Pembrolizumab	242	73 ± 2.75	76.0	NA	NA	200	NA	6
				B	Pembrolizumab, Lenvatinib	245	74 ± 3.25	69.0	NA	NA	203	NA	
Rosenberg 2023 (50)	USA	RCT	NCT03459846	A	Durvalumab	76	72 ± 10.75	72.0	14	34	28	0	4
				B	Durvalumab, Olaparib	78	79 ± 10.50	72.0	12	30	34	2	
Sonpavde 2023 (53)	USA	single-arm study	NA	B	Atezolizumab, CV301	19	78 ± 5.75	84.0	10	8	1	0	13
Donnell 2023 (49)	USA	RCT	NCT03288545	B	Pembrolizumab, Enfortumab Vedotin	76	71 ± 10.00	71.1	33	33	10	0	5

Subgroup A, patients received PD-1/L1 inhibitors monotherapy; Subgroup B, patients received PD-1/L1 dual immunotherapy; NA, not available; RCT, randomized controlled trial.

### 3.3 Radiographic response (CR, PR, SD, PD, ORR, DCR and DOR)

**Figure 2:**
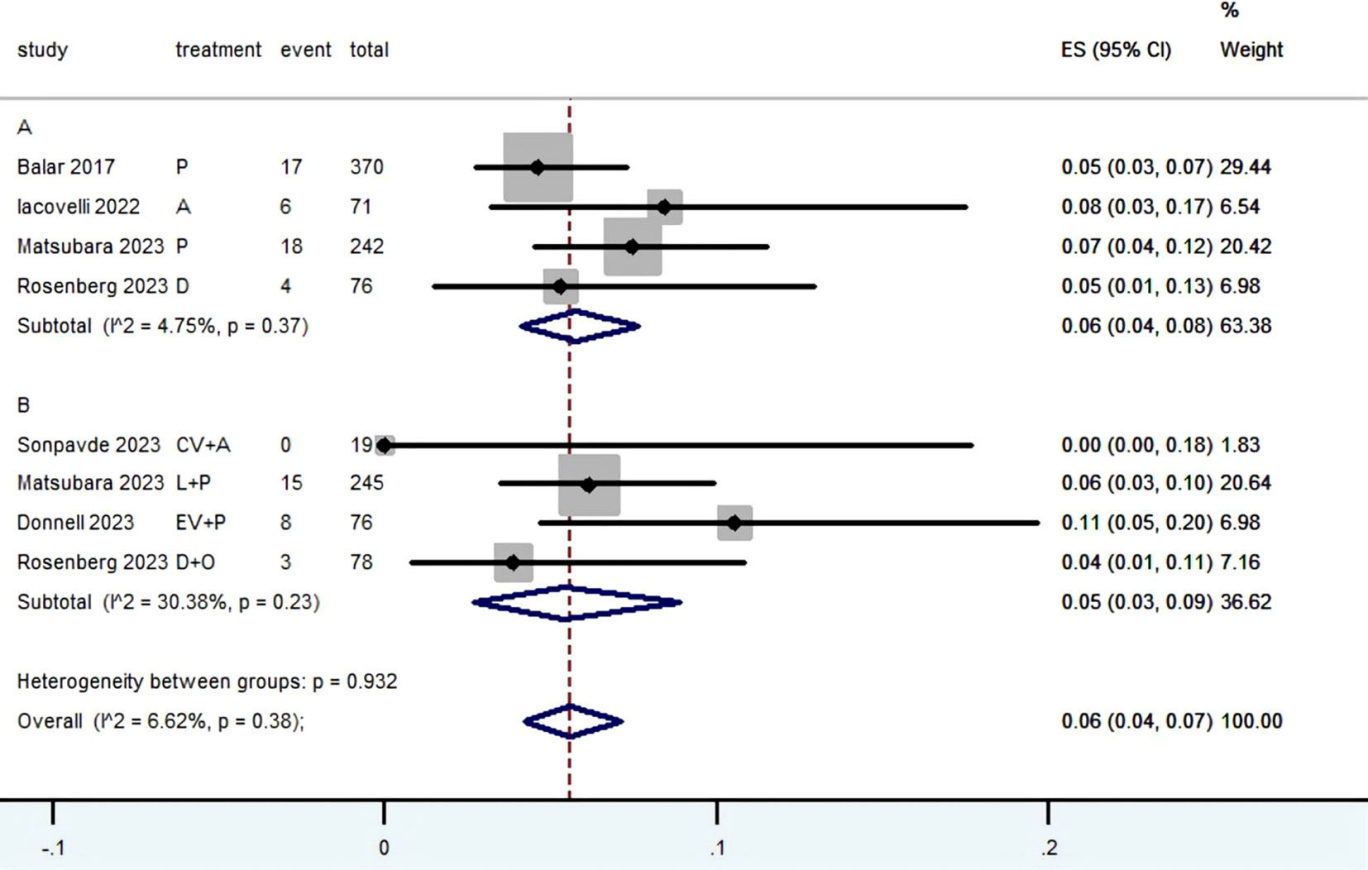
Forest plot of the meta-analysis for CR (Subgroup **A**: patients received PD-1/L1 inhibitors monotherapy; Subgroup **B**: patients received PD-1/L1 dual immunotherapy; P, Pembrolizumab; A, Avelumab; D, Durvalumab; CV, CV301; L, Lenvatinib; EV, Enfortumab Vedotin; O, Olaparib).

**Figure 3:**
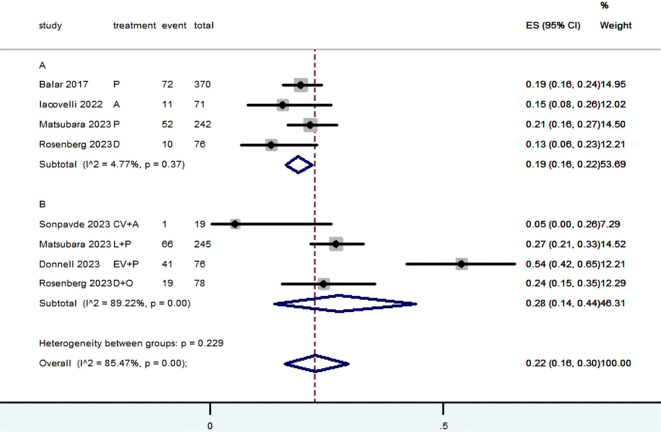
Forest plot of the meta-analysis for PR (Subgroup **A**: patients received PD-1/L1 inhibitors monotherapy; Subgroup **B**: patients received PD-1/L1 dual immunotherapy; P, Pembrolizumab; A, Avelumab; D, Durvalumab; CV, CV301; L, Lenvatinib; EV, Enfortumab Vedotin; O, Olaparib).

**Figure 4:**
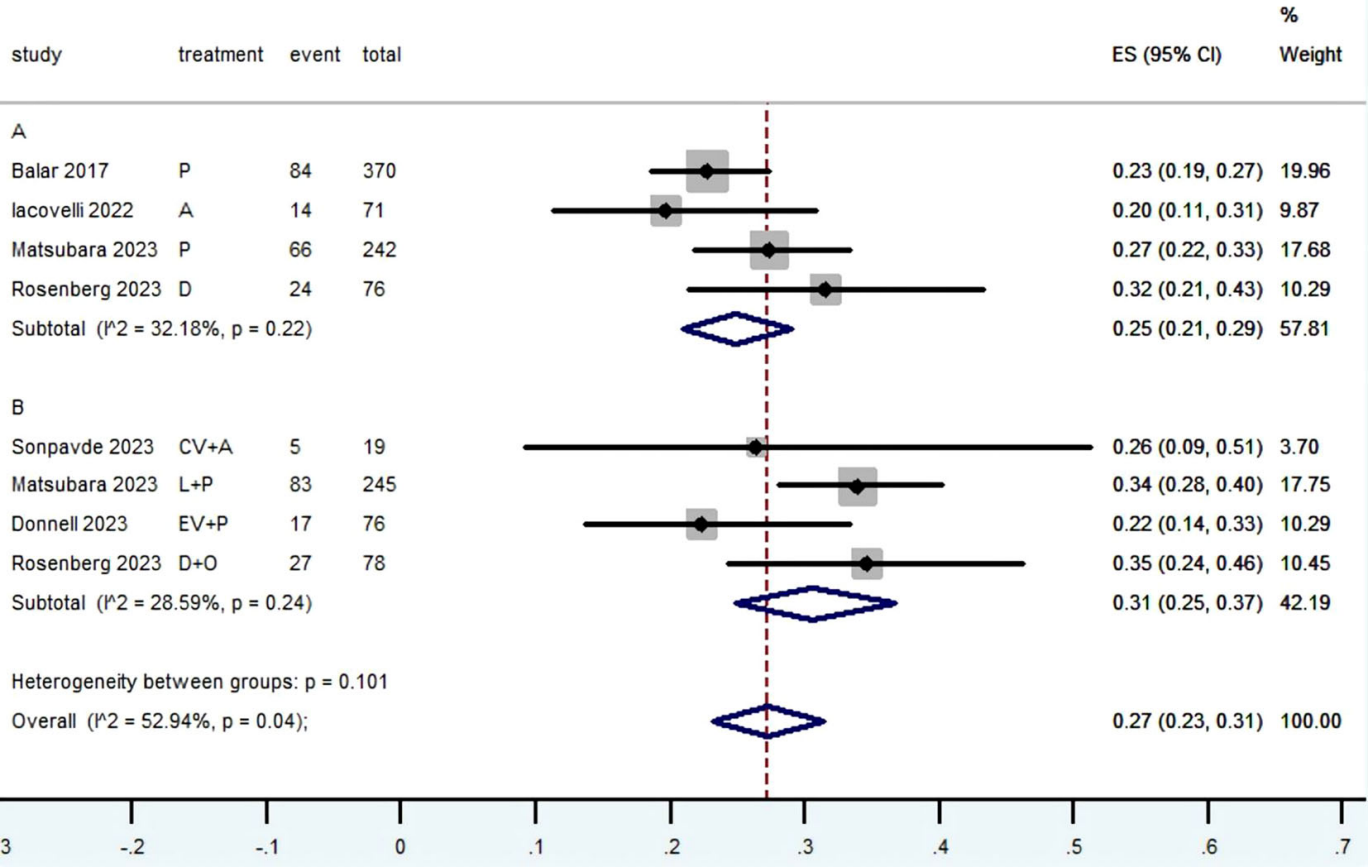
Forest plot of the meta-analysis for SD (Subgroup **A**: patients received PD-1/L1 inhibitors monotherapy; Subgroup **B**: patients received PD-1/L1 dual immunotherapy; P, Pembrolizumab; A, Avelumab; D, Durvalumab; CV, CV301; L, Lenvatinib; EV, Enfortumab Vedotin; O, Olaparib).

**Figure 5:**
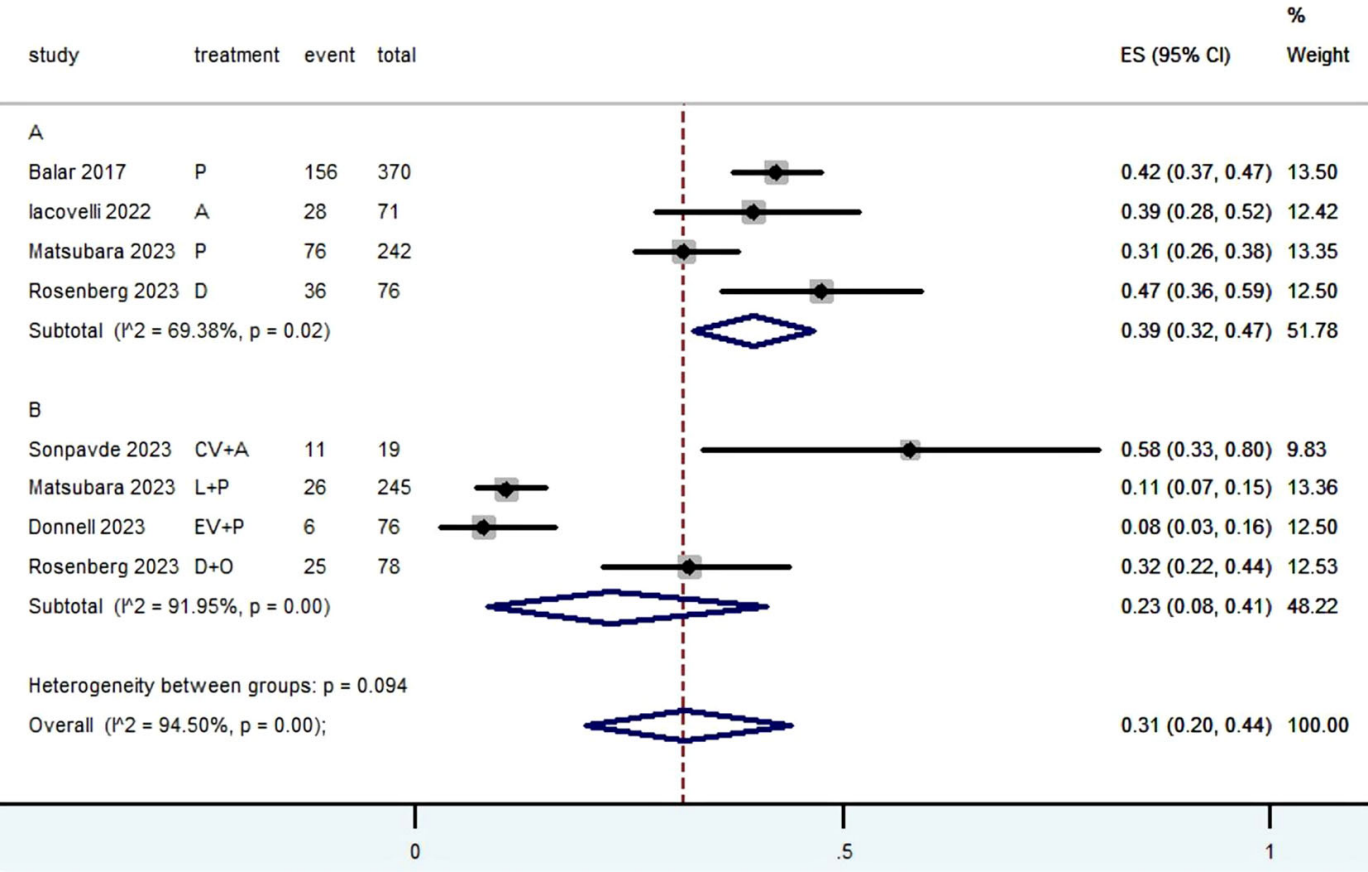
Forest plot of the meta-analysis for PD (Subgroup **A**: patients received PD-1/L1 inhibitors monotherapy; Subgroup **B**: patients received PD-1/L1 dual immunotherapy; P, Pembrolizumab; A, Avelumab; D, Durvalumab; CV, CV301; L, Lenvatinib; EV, Enfortumab Vedotin; O, Olaparib).

**Figure 6:**
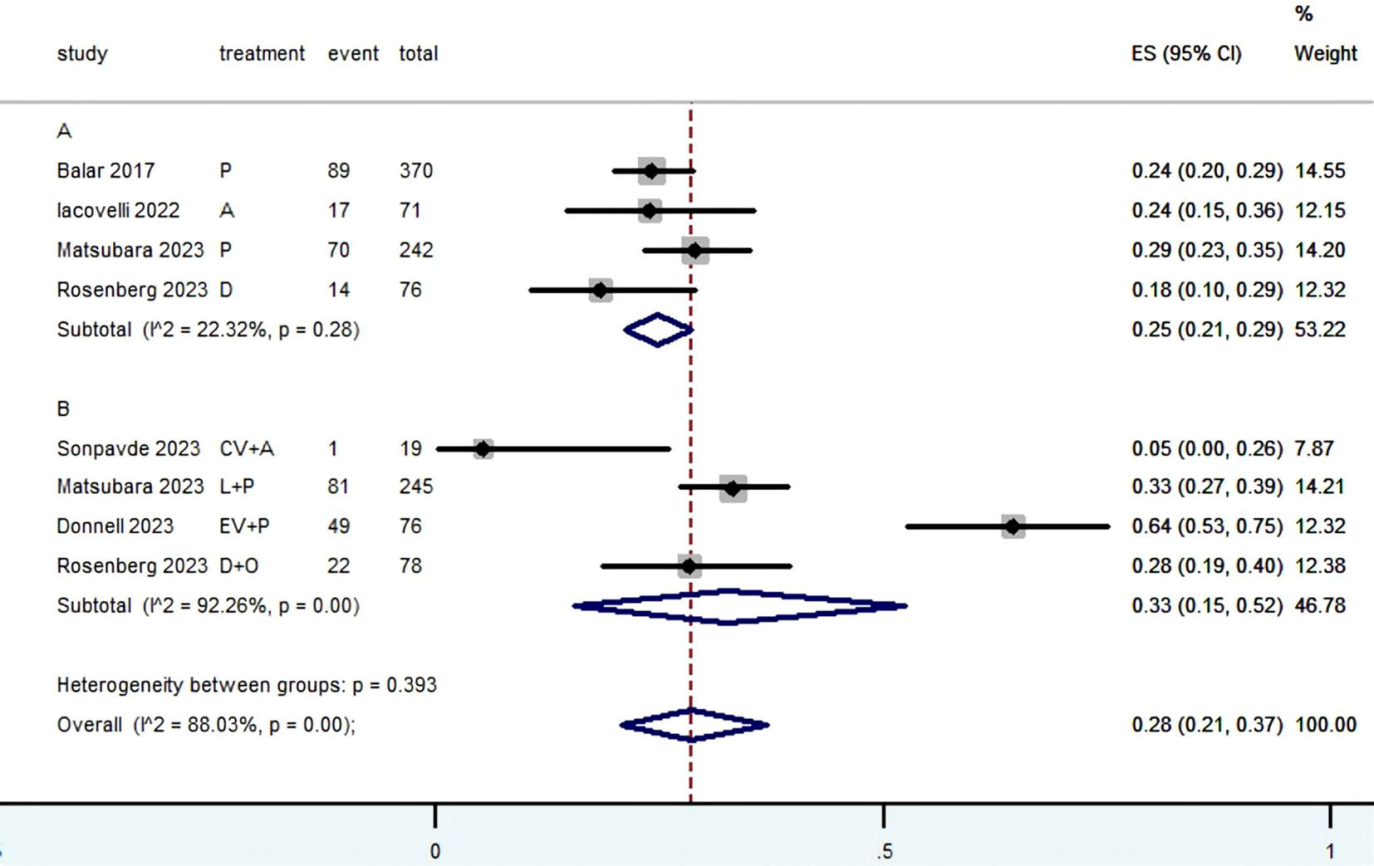
Forest plot of the meta-analysis for ORR (Subgroup **A**: patients received PD-1/L1 inhibitors monotherapy; Subgroup **B**: patients received PD-1/L1 dual immunotherapy; P, Pembrolizumab; A, Avelumab; D, Durvalumab; CV, CV301; L, Lenvatinib; EV, Enfortumab Vedotin; O, Olaparib).

**Figure 7:**
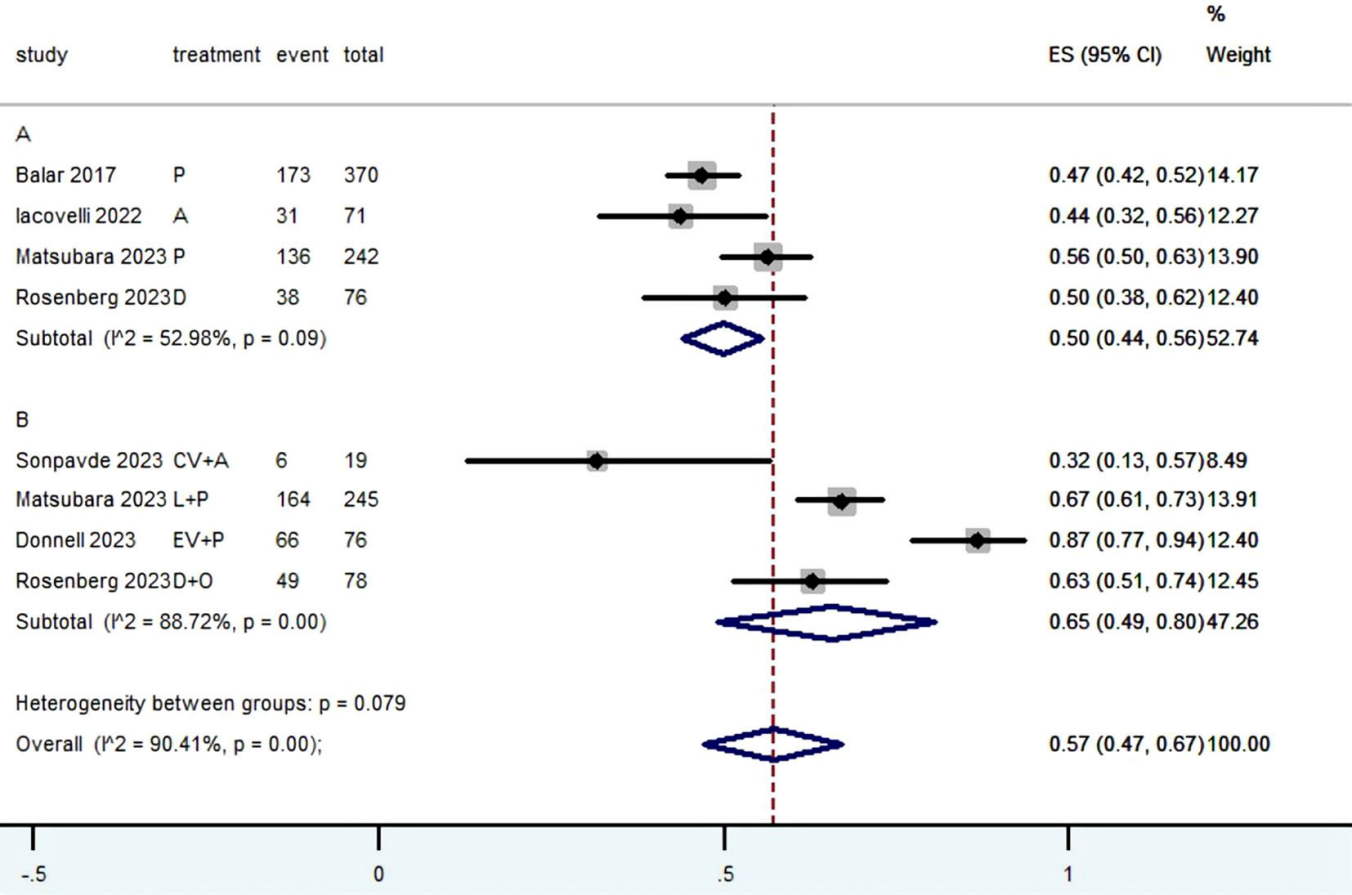
Forest plot of the meta-analysis for DCR (Subgroup **A**: patients received PD-1/L1 inhibitors monotherapy; Subgroup **B**: patients received PD-1/L1 dual immunotherapy; P, Pembrolizumab; A, Avelumab; D, Durvalumab; CV, CV301; L, Lenvatinib; EV, Enfortumab Vedotin; O, Olaparib).

**Table 2** provided a concise overview of the radiographic response outcomes. The radiographic response for patients with mUC who received PD-1/L1 inhibitors as first-line treatment were as follows: CR (0.06, 95% CI, 0.04 to 0.07) (**Figure 2**), PR (0.22, 95% CI, 0.16 to 0.30) (**Figure 3**), SD (0.27, 95% CI, 0.23 to 0.31) (**Figure 4**), PD (0.31, 95% CI, 0.20 to 0.44) (**Figure 5**), ORR (0.28, 95% CI, 0.21 to 0.37) (**Figure 6**) and DCR (0.57, 95% CI, 0.47 to 0.67) (**Figure 7**). In subgroup analysis, the radiographic response of patients with mUC who were treated with PD-1/L1 inhibitors monotherapy as the first-line treatment was as follows: CR (0.06, 95% CI, 0.04 to 0.08) (**Figure 2**), PR (0.19, 95% CI, 0.16 to 0.22) (**Figure 3**), SD (0.25, 95% CI, 0.21 to 0.29) (**Figure 4**), PD (0.39, 95% CI, 0.32 to 0.47) (**Figure 5**), ORR (0.25, 95% CI, 0.21 to 0.29) (**Figure 6**) and DCR (0.50, 95% CI, 0.44 to 0.56) (**Figure 7**). On the other hand, the radiographic response of patients with mUC who received PD-1/L1 dual immunotherapy as their first-line treatment was as follows: CR (0.05, 95% CI, 0.03 to 0.09) (**Figure 2**), PR (0.28, 95% CI, 0.14 to 0.44) (**Figure 3**), SD (0.31, 95% CI, 0.25 to 0.37) (**Figure 4**), PD (0.23, 95% CI, 0.08 to 0.41) (**Figure 5**), ORR (0.33, 95% CI, 0.15 to 0.52) (**Figure 6**) and DCR (0.65, 95% CI, 0.49 to 0.80) (**Figure 7**).

**Table 2:** The results of the meta-analysis for pCR, pPR, SD, DCR, PD, ORR and Grade≥ 3 TRAEs rate.

Outcomes	Patients	Heterogeneity		Overall effect size	95% CI of overall effect	Weight (%)
		I^2^ (%)	p-value			
CR						
Subgroup A	45	4.75	0.37	0.06	0.04-0.08	63.38
Subgroup B	26	30.38	0.23	0.05	0.03-0.09	36.62
Overall pooled CR	71	6.62	0.38	0.06	0.04-0.07	100.00
PR						
Subgroup A	145	4.77	0.37	0.19	0.16-0.22	53.69
Subgroup B	127	89.22	0.00	0.28	0.14-0.44	46.31
Overall pooled PR	272	85.47	0.00	0.22	0.16-0.30	100.00
SD						
Subgroup A	188	32.18	0.22	0.25	0.21-0.29	57.81
Subgroup B	132	28.59	0.24	0.31	0.25-0.37	42.19
Overall pooled SD	320	52.94	0.04	0.27	0.23-0.31	100.00
PD						
Subgroup A	296	69.38	0.02	0.39	0.32-0.47	51.78
Subgroup B	68	91.95	0.00	0.23	0.08-0.41	48.22
Overall pooled PD	364	94.50	0.00	0.31	0.20-0.44	100.00
ORR						
Subgroup A	190	22.32	0.28	0.25	0.21-0.29	53.22
Subgroup B	153	92.26	0.00	0.33	0.15-0.52	46.78
Overall pooled ORR	343	88.03	0.00	0.28	0.21-0.37	100.00
DCR						
Subgroup A	378	52.98	0.09	0.50	0.44-0.56	52.74
Subgroup B	285	88.72	0.00	0.65	0.49-0.80	47.26
Overall pooled DCR	663	90.41	0.00	0.57	0.47-0.67	100.00
Grade≥ 3 TRAEs rate						
Subgroup A	137	87.00	0.00	0.15	0.08-0.24	51.43
Subgroup B	190	92.73	0.00	0.40	0.21-0.61	48.57
Overall pooled Grade≥ 3 TRAEs rate	327	95.78	0.00	0.26	0.14-0.40	100.00

Subgroup A, patients received PD-1/L1 inhibitors monotherapy; Subgroup B, patients received PD-1/L1 dual immunotherapy; CR, complete response; PR, partial response; SD, stable disease; PD, progressive disease; ORR, objective response rate; DCR, disease control rate; TRAE, treatment-related adverse event.

### 3.4 PFS and OS

**Figure 8:**
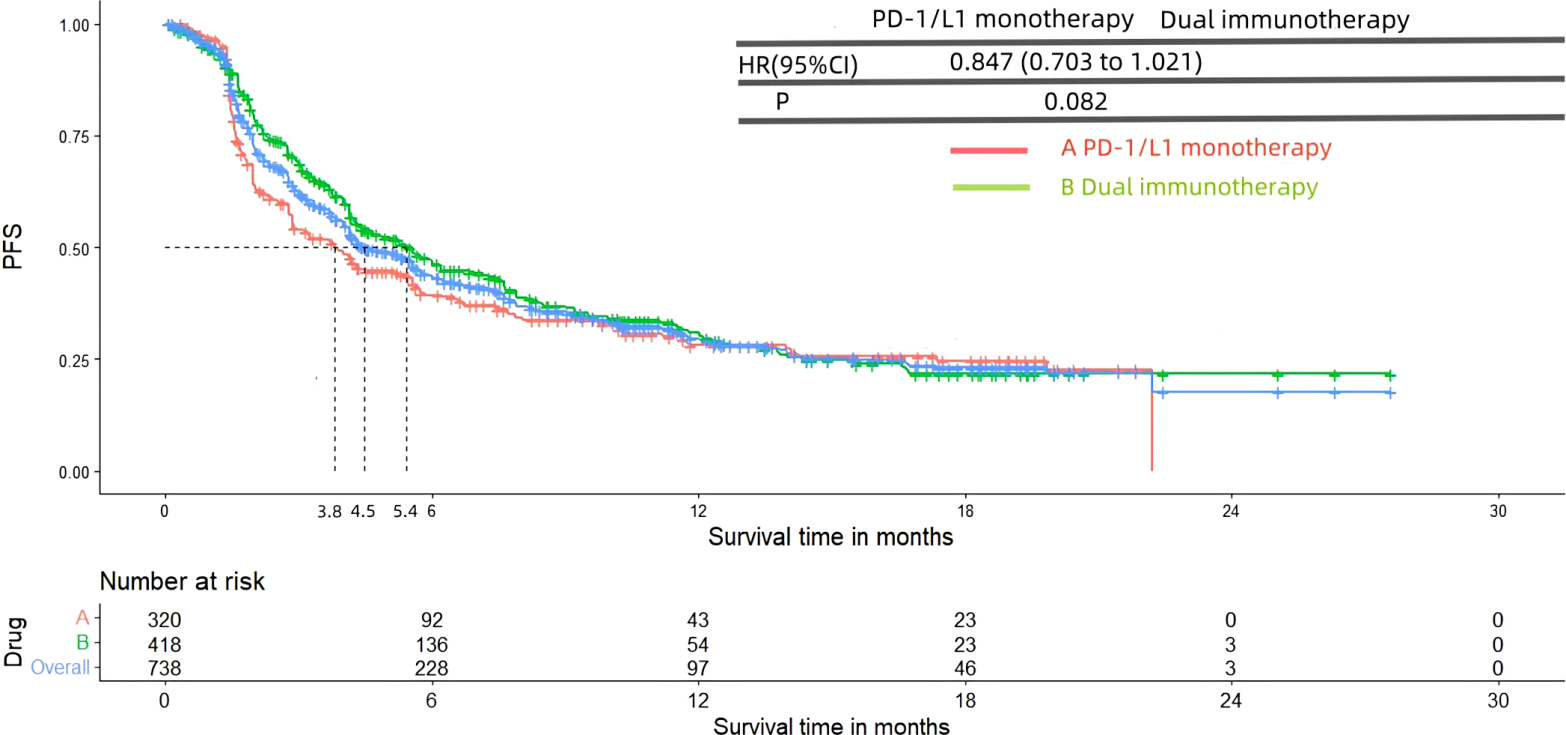
Kaplan-Meier curves for PFS (Subgroup **A**: patients received PD-1/L1 inhibitors monotherapy; Subgroup **B**: patients received PD-1/L1 dual immunotherapy).

**Figure 9:**
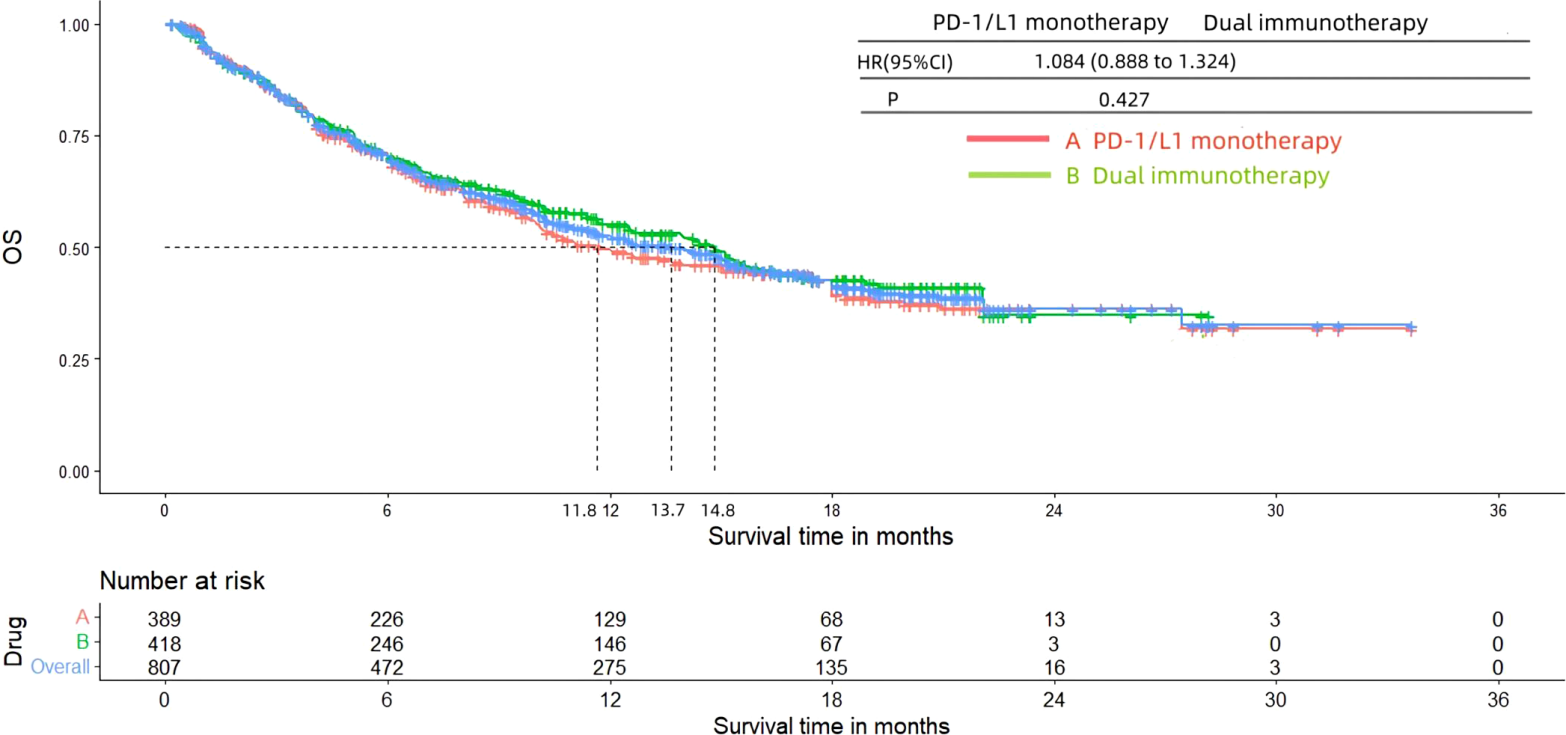
Kaplan-Meier curves for OS (Subgroup **A**: patients received PD-1/L1 inhibitors monotherapy; Subgroup **B**: patients received PD-1/L1 dual immunotherapy).

**Figures 8** and **9** presented the PFS and OS results of patients diagnosed with advanced or metastatic urothelial carcinoma, who were not eligible for platinum-based chemotherapy, and received PD-1/L1 inhibitors as their first-line treatment. Following the reconstruction of the cohort, we conducted an additional evaluation of PFS and OS using a Kaplan-Meier curve. Significantly, the median PFS and OS were 4.5 months and 13.7 months, respectively. In subgroup analysis, there was no significant difference in terms of the PFS (3.8 months vs 5.4 months, HR= 0.847, 95% Cl: 0.703 to 1.021, P= 0.082) and OS (11.8 months vs 14.8 months, HR= 1.084, 95% Cl: 0.888 to 1.324, P= 0.427) Kaplan-Meier curve between two groups.

### 3.5 Grade≥ 3 TRAEs rate

**Figure 10:**
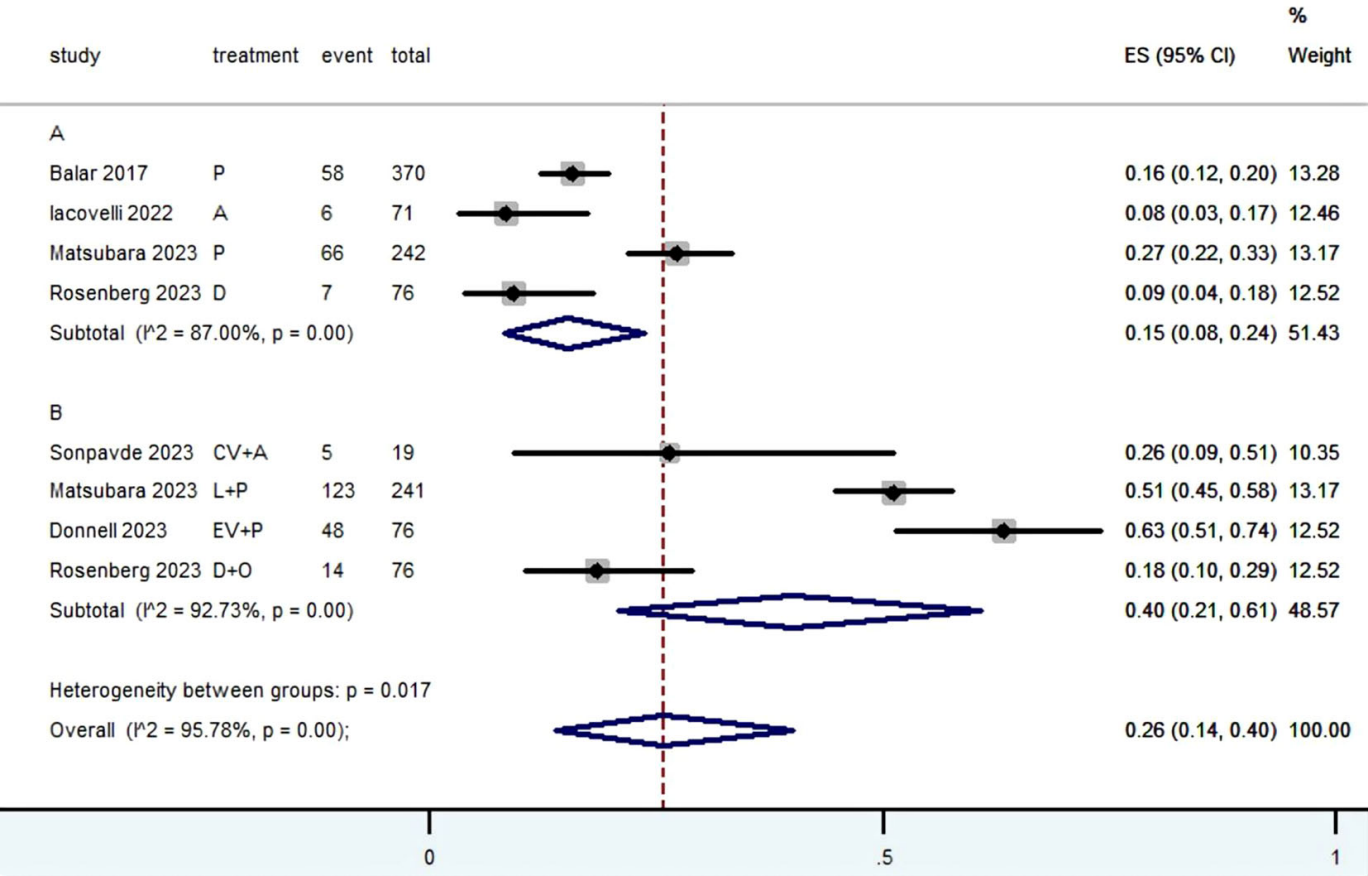
Forest plot of the meta-analysis for Grade≥ 3 TRAEs rate(Subgroup **A**: patients received PD-1/L1 inhibitors monotherapy; Subgroup **B**: patients received PD-1/L1 dual immunotherapy).

The Grade≥ 3 TRAEs rate was found to be 0.26 (95% CI, 0.14 to 0.40) (**Table 2**; **Figure 10**) among patients with mUC who received PD-1/L1 inhibitors as their first-line treatment. In subgroup analysis, The Grade≥ 3 TRAEs rate in PD-1/L1 inhibitors monotherapy and PD-1/L1 dual immunotherapy were 0.15 (95% CI, 0.08 to 0.24) and 0.40 (95% CI, 0.21 to 0.61), respectively. The most prevalent grade 3–5 AEs associated with PD-1/L1 dual immunotherapy were hypertension (10.7%), proteinuria (6.8%), lipase level increased (5.1%), diarrhea (3.9%), fatigue (3.9%), rash maculopapular (3.2%), anemia (2.4%), peripheral neuropathy (2.2%) and neutropenia (2.2%).

## 4 Discussion

First-line treatment with PD-1/L1 inhibitors has demonstrated substantial therapeutic advantages in patients with advanced or metastatic urothelial carcinoma who had previously received cisplatin therapy (32, 54–56). Nevertheless, a considerable percentage of patients do not meet the criteria for platinum chemotherapy, and further research is needed to determine the therapeutic advantages of PD-1/L1 inhibitors in patients who cannot tolerate platinum-based treatments. Currently, an increasing number of clinical trials have assessed the safety and effectiveness of PD-1/L1 inhibitors in patients who were not responsive to platinum-based chemotherapy. This study involved a systematic review and meta-analysis to assess the effectiveness and safety of PD-1/L1 inhibitors as the first-line treatment for patient with advanced or metastatic urothelial carcinoma who could not be treated with platinum-based chemotherapy.

Cisplatin-based chemotherapy is currently the recommended regimen for first-line treatment in patients with mUC. However, around 40% of patients are unable to undergo these treatment regimens due to renal impairment, low performance status, or other comorbidities (12, 57, 58). Carboplatin in combination with gemcitabine is presently a standard option for patients who are unable to tolerate cisplatin. However, this treatment is linked to a low percentage of positive response (about 36%-42%), a short duration of response (6.3-7.1 months), a median OS of nine months and somewhat unfavorable tolerability (9, 59, 60). Therefore, there is still a significant need for a highly effective initial treatment option that produces quick and long-lasting responses and has a tolerable safety profile. This will ensure that a greater number of individuals with advanced or metastatic urothelial carcinoma who cannot tolerate cisplatin treatment can obtain long-lasting therapeutic benefits.

Targeting the PD-1/L1 pathway with immune checkpoint inhibitors (ICIs) has shown encouraging results (61). A number of ICIs have been approved as first-line treatment in case of cisplatin-ineligible patients or as second-line treatment for patients with mUC, and about 30% of patients with mUC could respond to ICIs immunotherapy (62). Currently, five ICIs, including two anti-PD-1 antibodies (Pembro and nivolumab) and three anti-PD-L1 antibodies (atezolizumab, avelumab and durvalumab), have been granted approval by the US Food and Drug Administration for patients with unresectable or metastatic urothelial carcinoma who recurred or progressed after platinum-based chemotherapy (63).

Our results revealed that PD-1/L1 inhibitors had certain therapeutic advantage, with ORR of 28% and DCR of 57%. Besides, the median PFS and OS were 4.5 and 13.7 months, respectively. In addition, PD-1/L1 inhibitors showed an acceptable level of safety, with TRAEs≥ grade 3 rates of 0.26. Therefore, our finding suggests that PD-1/L1 inhibitors may be a feasible and safe first-line treatment for patients who are not suitable for platinum-based chemotherapy. The current meta-analysis included a total of six studies, including three randomized controlled trials and three single-arm trials. Due to differences in study type, the analysis was limited to patients with mUC who were not eligible for platinum-based chemotherapy and received PD-1/L1 inhibitors as first-line treatment. Subgroup analysis was performed to compare the efficacy and safety of PD-1/L1 inhibitors monotherapy versus PD-1/L1 dual immunotherapy. There were four PD-1/L1 dual immunotherapy regimens: Pembro plus lenvatinib, durvalumab plus olaparib, atezolizumab plus CV301, and Pembro plus EV (**Table 1**). Our results showed that PD-1/L1 inhibitors monotherapy had an ORR rate of 25% and a DCR rate of 50%, while PD-1/L1 dual immunotherapy had a better ORR rate of 33% and a DCR rate of 65%. The better ORR and DCR demonstrated the additive or synergistic antitumor efficacy of PD-1/L1 dual immunotherapy. Besides, PD-1/L1 dual immunotherapy provided longer PFS and OS compared with PD-1/L1 inhibitors monotherapy, though there was no significant difference. The findings revealed that PD-1/L1 dual immunotherapy might be a more effective regimen than PD-1/L1 inhibitors monotherapy. The non-significant results regarding OS and PFS may be affected by sample size or heterogeneity. Among the four PD-1/L1 dual immunotherapy regimens included, it is important to highlight that the combination of EV+Pembro (49) in EV-103 study demonstrated a manageable safety profile and yielded a high ORR (64.5%) with long-lasting responses. The ORR observed in this study were consistent across a range of prespecified subgroups, including patients with liver metastases, and antitumor activity was seen regardless of PD-L1 status. Additionally, the treatment showed a median OS of 22.3 months and the median PFS was not reached. The numerical outcomes of these results were significantly better than those observed in trials involving the current standard treatment (gemcitabine + carboplatin, or PD-1/L1 inhibitors monotherapy), specifically for patients who were not eligible for cisplatin (9, 32, 51, 52, 59, 60). The data indicated that the combination of EV plus Pembro could be a promising new treatment option for individuals with advanced bladder cancer who are unable to receive cisplatin as their first-line therapy. EV is an ADC that consists of a completely human monoclonal antibody that targets nectin-4 and monomethyl auristatin E (9). EV transports monomethyl auristatin E to cells that express nectin-4, resulting in the interruption of the cell cycle and subsequent cell demise. Both EV and Pembro, as standalone therapies, have demonstrated overall survival advantages relative to second-line or third-line treatments in patients with mUC (32, 64, 65). Preclinical results indicate that vedotin ADCs, particularly EV, combined with PD-1/L1 inhibitors like Pembro, may augment anticancer activity in relation to their respective modes of action and demonstrate complementary efficacy (43–45).

Regarding safety, the Grade≥ 3 TRAEs rate of PD-1/L1 dual immunotherapy was significantly higher than that of PD-1/L1 inhibitors monotherapy. The most prevalent TRAEs associated with PD-1/L1 dual immunotherapy included hypertension, proteinuria, lipase level increased, diarrhea, fatigue, rash maculopapular, anemia, peripheral neuropathy and neutropenia. It is advisable to formulate clinical response strategies for these high-frequency or severe AEs to offer more thorough guidance for clinical applications. Arjun V et al. reported that most immune-mediated adverse events resolved with corticosteroid treatment and without sequelae (32). Peter H et al (49) reported that TRAEs could be managed with EV treatment interruption, dose reduction, treatment discontinuation (EV and/or Pembro), and/or corticosteroids. The safety outcomes underscore the necessity of educating both healthcare professionals and patients; prompt intervention for adverse events is crucial for effectively treating patients with the combination.

Our study has its advantage. This study was the first meta-analysis to assess the effectiveness and safety of PD-1/L1 inhibitors as first-line treatment for patients with advanced or metastatic urothelial carcinoma who were ineligible for platinum-based chemotherapy. Furthermore, the IPDformKM software package was utilized to reconstruct Kaplan-Meier curves for OS and PFS. This was done in order to display the tumor results in a concise and comprehensible manner. Undoubtedly, our study has certain limitations. Initially, the size of the sample was relatively limited. The analysis included only six trials. In addition, the utilization of single-arm clinical studies resulted in indirect comparisons across various treatment alternatives. Moreover, the six studies exhibited considerable heterogeneity with regards to their study methodologies, patient demographics, and treatment regimens. Furthermore, certain trials have not yet reached their end-points, such as PFS. Hence, it is imperative to exercise caution when interpreting our findings. In order to establish the safety and effectiveness of PD-1/L1 inhibitors as a first-line treatment for mUC patients who are unable to undergo platinum-based chemotherapy, it is crucial to carry out more multicenter, randomized controlled trials and prolong the period of follow-up. Evaluating the role of biomarkers such as PD-L1 status may help predict response to PD-1/L1 dual immunotherapy.

In conclusion, our results indicated that PD-1/L1 inhibitors as first-line treatment were feasible and safe for patients with advanced or metastatic urothelial carcinoma who are ineligible for platinum-based chemotherapy. Specially, the combination of EV and Pembro demonstrated superior therapeutic efficacy compared to trials utilizing the current standard treatment, suggesting it as a potentially attractive alternative treatment approach.

## Data Availability

The original contributions presented in the study are included in the article/**Supplementary Materials**, further inquiries can be directed to the corresponding author/s.
