# Supplementary material for: Effectiveness and safety of PD-1/L1 inhibitors as first-line therapy for patients with advanced or metastatic urothelial carcinoma who are ineligible for platinum-based chemotherapy: a meta-analysis
Source: Front Immunol. 2025 Feb 12;16:1430673. doi: 10.3389/fimmu.2025.1430673 (PMC11860080; doi:10.3389/fimmu.2025.1430673)
Supplement: Supplementary file 1 [file Table1.docx]

| **Cochrane library** | | |
| --- | --- | --- |
| No. | Query | Results |
| 1 | (PD-1Inhibitors OR PD-1 Inhibitors OR PD 1 Inhibitors OR PD-1 Inhibitor OR Inhibitor, PD-1 OR PD 1 Inhibitor OR Programmed Cell Death Protein 1 Inhibitor OR Programmed Cell Death Protein 1 Inhibitors OR Nivolumab OR Opdivo OR ONO-4538 OR ONO 4538 OR ONO4538 OR MDX-1106 OR MDX 1106 OR MDX1106 OR BMS-936558 OR BMS 936558 OR BMS936558 OR Pembrolizumab OR SCH-900475 OR lambrolizumab OR MK-3475 OR Keytruda OR Tislelizumab OR BGB-A317 OR Toripalimab OR Camrelizumab OR carrelizumab OR SHR-1210 OR SHR 1210 OR Sintilimab OR IBI 308 OR IBI308 OR IBI-308 OR Zimberelimab OR GLS-010 OR Prolgolimab OR Dostarlimab OR Jemperli OR dostarlimab-gxly OR TSR-042 OR GSK4057190 OR PD-L1 Inhibitors OR PD-L1 Inhibitors OR PD L1 Inhibitors OR PD-L1 Inhibitor OR PD L1 Inhibitor OR Programmed Death-Ligand 1 Inhibitors OR Programmed Death Ligand 1 Inhibitors OR PD-1 PD-L1 Blockade OR Blockade, PD-1 PD-L1 OR PD 1 PD L1 Blockade OR Atezolizumab OR anti-PDL1 OR immunoglobulin G1, anti-(human CD antigen CD274) (human monoclonal MDPL3280a heavy chain), disulfide with human monoclonal MDPL3280a kappa-chain, dimer OR MPDL3280A OR MPDL-3280A OR Tecentriq OR RG7446 OR RG-7446 OR Durvalumab OR MEDI4736 OR MEDI-4736 OR Imfinzi OR Avelumab OR MSB-0010682 OR MSB0010718C OR MSB-0010718C OR MSB0010682 OR bavencio):ti,ab,kw | 13948 |
| 2 | ((urothelial carcinoma) OR(Carcinomas,Transitional Cell) OR(Cell Carcinoma, Transitional) OR(Cell Carcinomas, Transitional) OR(Transitional Cell Carcinoma) OR(Transitional Cell Carcinomas) OR carcinoma of renal calyx OR renal pelvic carcinoma OR renal pelvis cancer OR carcinoma of renal pelvis OR Upper tract urothelial carcinoma OR UTUC OR renal pelvic OR Malignant tumor of the ureter OR eoplasm, Ureteral OR Ureteral Neoplasm OR Neoplasms, Ureteral OR Ureter Neoplasms OR Ureter Neoplasm OR Neoplasms of Ureter OR Cancer of Ureter OR Ureter Cancers OR Ureter, Cancer Of OR Ureter Cancer OR Ureteral Cancer OR Cancer, Ureteral OR Cancers, Ureteral OR Ureteral Cancers OR Cancer of the Ureter OR Urinary Bladder Neoplasms OR Neoplasm, Urinary Bladder OR Urinary Bladder Neoplasm OR Bladder Tumors OR Bladder Tumor OR Tumor, Bladder OR Tumors, Bladder OR Neoplasms, Bladder OR Bladder Neoplasms OR Bladder Neoplasm OR Neoplasm, Bladder OR Urinary Bladder Cancer OR Cancer, Urinary Bladder OR Malignant Tumor of Urinary Bladder OR Cancer of the Bladder OR Bladder Cancer OR Bladder Cancers OR Cancer, Bladder OR Cancer of Bladder):ti,ab,kw | 7776 |
| 3 | (randomized controlled tria OR controlled clinical trial OR randomized OR placebo OR clinical trials as topic OR randomly OR trial):ti,ab,kw | 1476510 |
| 4 | 1 AND 2 AND 3 | 642 |

| **Web of science** | | |
| --- | --- | --- |
| No. | Query | Results |
| 1 | TI=(PD-1Inhibitors OR PD-1 Inhibitors OR PD 1 Inhibitors OR PD-1 Inhibitor OR Inhibitor, PD-1 OR PD 1 Inhibitor OR Programmed Cell Death Protein 1 Inhibitor OR Programmed Cell Death Protein 1 Inhibitors OR Nivolumab OR Opdivo OR ONO-4538 OR ONO 4538 OR ONO4538 OR MDX-1106 OR MDX 1106 OR MDX1106 OR BMS-936558 OR BMS 936558 OR BMS936558 OR Pembrolizumab OR SCH-900475 OR lambrolizumab OR MK-3475 OR Keytruda OR Tislelizumab OR BGB-A317 OR Toripalimab OR Camrelizumab OR carrelizumab OR SHR-1210 OR SHR 1210 OR Sintilimab OR IBI 308 OR IBI308 OR IBI-308 OR Zimberelimab OR GLS-010 OR Prolgolimab OR Dostarlimab OR Jemperli OR dostarlimab-gxly OR TSR-042 OR GSK4057190 OR PD-L1 Inhibitors OR PD-L1 Inhibitors OR PD L1 Inhibitors OR PD-L1 Inhibitor OR PD L1 Inhibitor OR Programmed Death-Ligand 1 Inhibitors OR Programmed Death Ligand 1 Inhibitors OR PD-1 PD-L1 Blockade OR Blockade, PD-1 PD-L1 OR PD 1 PD L1 Blockade OR Atezolizumab OR anti-PDL1 OR immunoglobulin G1, anti-(human CD antigen CD274) (human monoclonal MDPL3280a heavy chain), disulfide with human monoclonal MDPL3280a kappa-chain, dimer OR MPDL3280A OR MPDL-3280A OR Tecentriq OR RG7446 OR RG-7446 OR Durvalumab OR MEDI4736 OR MEDI-4736 OR Imfinzi OR Avelumab OR MSB-0010682 OR MSB0010718C OR MSB-0010718C OR MSB0010682 OR bavencio) | 26933 |
| 2 | TI=((urothelial carcinoma) OR(Carcinomas,Transitional Cell) OR(Cell Carcinoma, Transitional) OR(Cell Carcinomas, Transitional) OR(Transitional Cell Carcinoma) OR(Transitional Cell Carcinomas) OR carcinoma of renal calyx OR renal pelvic carcinoma OR renal pelvis cancer OR carcinoma of renal pelvis OR Upper tract urothelial carcinoma OR UTUC OR renal pelvic OR Malignant tumor of the ureter OR eoplasm, Ureteral OR Ureteral Neoplasm OR Neoplasms, Ureteral OR Ureter Neoplasms OR Ureter Neoplasm OR Neoplasms of Ureter OR Cancer of Ureter OR Ureter Cancers OR Ureter, Cancer Of OR Ureter Cancer OR Ureteral Cancer OR Cancer, Ureteral OR Cancers, Ureteral OR Ureteral Cancers OR Cancer of the Ureter OR Urinary Bladder Neoplasms OR Neoplasm, Urinary Bladder OR Urinary Bladder Neoplasm OR Bladder Tumors OR Bladder Tumor OR Tumor, Bladder OR Tumors, Bladder OR Neoplasms, Bladder OR Bladder Neoplasms OR Bladder Neoplasm OR Neoplasm, Bladder OR Urinary Bladder Cancer OR Cancer, Urinary Bladder OR Malignant Tumor of Urinary Bladder OR Cancer of the Bladder OR Bladder Cancer OR Bladder Cancers OR Cancer, Bladder OR Cancer of Bladder) | 65231 |
| 3 | TI=(randomized controlled tria OR controlled clinical trial OR randomized OR placebo OR clinical trials as topic OR randomly OR trial) | 627386 |
| 4 | 1 AND 2 AND 3 | 277 |

| **Embase** | | |
| --- | --- | --- |
| No. | Query | Results |
| 1 | ('pd-1inhibitors':ab,ti OR 'pd-1 inhibitors':ab,ti OR 'pd 1 inhibitors':ab,ti OR 'pd-1 inhibitor':ab,ti OR 'inhibitor, pd-1':ab,ti OR 'pd 1 inhibitor':ab,ti OR 'programmed cell death protein 1 inhibitor':ab,ti OR 'programmed cell death protein 1 inhibitors':ab,ti OR 'nivolumab':ab,ti OR 'opdivo':ab,ti OR 'ono 4538':ab,ti OR 'ono4538':ab,ti OR 'mdx 1106':ab,ti OR 'mdx1106':ab,ti OR 'bms 936558':ab,ti OR 'bms936558':ab,ti OR 'pembrolizumab':ab,ti OR 'sch 900475':ab,ti OR 'lambrolizumab':ab,ti OR 'mk 3475':ab,ti OR 'keytruda':ab,ti OR 'tislelizumab':ab,ti OR 'bgb a317':ab,ti OR 'toripalimab':ab,ti OR 'camrelizumab':ab,ti OR 'carrelizumab':ab,ti OR 'shr 1210':ab,ti OR 'sintilimab':ab,ti OR 'ibi308':ab,ti OR 'ibi 308':ab,ti OR 'zimberelimab':ab,ti OR 'gls 010':ab,ti OR 'prolgolimab':ab,ti OR 'dostarlimab':ab,ti OR 'jemperli':ab,ti OR 'dostarlimab gxly':ab,ti OR 'tsr 042':ab,ti OR 'gsk4057190':ab,ti OR 'pd-l1 inhibitors':ab,ti OR 'pd l1 inhibitors':ab,ti OR 'pd-l1 inhibitor':ab,ti OR 'pd l1 inhibitor':ab,ti OR 'programmed death-ligand 1 inhibitors':ab,ti OR 'programmed death ligand 1 inhibitors':ab,ti OR 'pd-1 pd-l1 blockade':ab,ti OR 'blockade, pd-1 pd-l1':ab,ti OR 'pd 1 pd l1 blockade':ab,ti OR 'atezolizumab':ab,ti OR 'anti pdl1':ab,ti OR 'immunoglobulin g1, anti-':ab,ti) AND 'human cd antigen cd274':ab,ti AND 'human monoclonal mdpl3280a heavy chain':ab,ti AND 'disulfide with human monoclonal mdpl3280a kappa-chain, dimer':ab,ti OR 'mpdl3280a':ab,ti OR 'mpdl 3280a':ab,ti OR 'tecentriq':ab,ti OR 'rg7446':ab,ti OR 'rg 7446':ab,ti OR 'durvalumab':ab,ti OR 'medi4736':ab,ti OR 'medi 4736':ab,ti OR 'imfinzi':ab,ti OR 'avelumab':ab,ti OR 'msb 0010682':ab,ti OR 'msb0010718c':ab,ti OR 'msb 0010718c':ab,ti OR 'msb0010682':ab,ti OR 'bavencio':ab,ti | 5681 |
| 2 | 'urothelial carcinoma':ab,ti OR 'carcinomas,transitional cell':ab,ti OR 'cell carcinoma, transitional':ab,ti OR 'cell carcinomas, transitional':ab,ti OR 'transitional cell carcinoma':ab,ti OR 'transitional cell carcinomas':ab,ti OR 'carcinoma of renal calyx':ab,ti OR 'renal pelvic carcinoma':ab,ti OR 'renal pelvis cancer':ab,ti OR 'carcinoma of renal pelvis':ab,ti OR 'Upper tract urothelial carcinoma':ab,ti OR 'UTUC':ab,ti OR 'renal pelvic':ab,ti OR 'malignant tumor of the ureter':ab,ti OR 'eoplasm, ureteral':ab,ti OR 'ureteral neoplasm':ab,ti OR 'neoplasms, ureteral':ab,ti OR 'ureter neoplasms':ab,ti OR 'ureter neoplasm':ab,ti OR 'neoplasms of ureter':ab,ti OR 'cancer of ureter':ab,ti OR 'ureter cancers':ab,ti OR 'ureter, cancer of':ab,ti OR 'ureter cancer':ab,ti OR 'ureteral cancer':ab,ti OR 'cancer, ureteral':ab,ti OR 'cancers, ureteral':ab,ti OR 'ureteral cancers':ab,ti OR 'cancer of the ureter':ab,ti OR 'urinary bladder neoplasms':ab,ti OR 'neoplasm, urinary bladder':ab,ti OR 'urinary bladder neoplasm':ab,ti OR 'bladder tumors':ab,ti OR 'bladder tumor':ab,ti OR 'tumor, bladder':ab,ti OR 'tumors, bladder':ab,ti OR 'neoplasms, bladder':ab,ti OR 'bladder neoplasms':ab,ti OR 'bladder neoplasm':ab,ti OR 'neoplasm, bladder':ab,ti OR 'urinary bladder cancer':ab,ti OR 'cancer, urinary bladder':ab,ti OR 'malignant tumor of urinary bladder':ab,ti OR 'cancer of the bladder':ab,ti OR 'bladder cancer':ab,ti OR 'bladder cancers':ab,ti OR 'cancer, bladder':ab,ti OR 'cancer of bladder':ab,ti | 93401 |
| 3 | 'randomized controlled trial':ab,ti OR 'controlled clinical trial':ab,ti OR randomized:ab,ti OR placebo:ab,ti OR 'clinical trials as topic':ab,ti OR randomly:ab,ti OR trial:ab,ti | 2156801 |
| 4 | 1 AND 2 AND 3 | 319 |

| **PubMed** | | |
| --- | --- | --- |
| No. | Query | Results |
| 1 | PD-1Inhibitors[Title/Abstract] OR PD-1 Inhibitors[Title/Abstract] OR PD 1 Inhibitors[Title/Abstract] OR PD-1 Inhibitor[Title/Abstract] OR Inhibitor, PD-1[Title/Abstract] OR PD 1 Inhibitor[Title/Abstract] OR Programmed Cell Death Protein 1 Inhibitor[Title/Abstract] OR Programmed Cell Death Protein 1 Inhibitors[Title/Abstract] OR Nivolumab[Title/Abstract] OR Opdivo[Title/Abstract] OR ONO-4538[Title/Abstract] OR ONO 4538[Title/Abstract] OR ONO4538[Title/Abstract] OR MDX-1106[Title/Abstract] OR MDX 1106[Title/Abstract] OR MDX1106[Title/Abstract] OR BMS-936558[Title/Abstract] OR BMS 936558[Title/Abstract] OR BMS936558[Title/Abstract] OR Pembrolizumab[Title/Abstract] OR SCH-900475[Title/Abstract] OR lambrolizumab[Title/Abstract] OR MK-3475[Title/Abstract] OR Keytruda[Title/Abstract] OR Tislelizumab[Title/Abstract] OR BGB-A317[Title/Abstract] OR Toripalimab[Title/Abstract] OR Camrelizumab[Title/Abstract] OR carrelizumab[Title/Abstract] OR SHR-1210[Title/Abstract] OR SHR 1210[Title/Abstract] OR Sintilimab[Title/Abstract] OR IBI 308[Title/Abstract] OR IBI308[Title/Abstract] OR IBI-308[Title/Abstract] OR Zimberelimab[Title/Abstract] OR GLS-010[Title/Abstract] OR Prolgolimab[Title/Abstract] OR Dostarlimab[Title/Abstract] OR Jemperli[Title/Abstract] OR dostarlimab-gxly[Title/Abstract] OR TSR-042[Title/Abstract] OR GSK4057190[Title/Abstract] OR PD-L1 Inhibitors[Title/Abstract] OR PD-L1 Inhibitors[Title/Abstract] OR PD L1 Inhibitors[Title/Abstract] OR PD-L1 Inhibitor[Title/Abstract] OR PD L1 Inhibitor[Title/Abstract] OR Programmed Death-Ligand 1 Inhibitors[Title/Abstract] OR Programmed Death Ligand 1 Inhibitors[Title/Abstract] OR PD-1 PD-L1 Blockade[Title/Abstract] OR Blockade, PD-1 PD-L1[Title/Abstract] OR PD 1 PD L1 Blockade[Title/Abstract] OR Atezolizumab[Title/Abstract] OR anti-PDL1[Title/Abstract] OR immunoglobulin G1, anti-(human CD antigen CD274) (human monoclonal MDPL3280a heavy chain), disulfide with human monoclonal MDPL3280a kappa-chain, dimer[Title/Abstract] OR MPDL3280A[Title/Abstract] OR MPDL-3280A[Title/Abstract] OR Tecentriq[Title/Abstract] OR RG7446[Title/Abstract] OR RG-7446[Title/Abstract] OR Durvalumab[Title/Abstract] OR MEDI4736[Title/Abstract] OR MEDI-4736[Title/Abstract] OR Imfinzi[Title/Abstract] OR Avelumab[Title/Abstract] OR MSB-0010682[Title/Abstract] OR MSB0010718C[Title/Abstract] OR MSB-0010718C[Title/Abstract] OR MSB0010682[Title/Abstract] OR bavencio[Title/Abstract] | 2301 |
| 2 | (urothelial carcinoma[Title/Abstract]) OR(Carcinomas,Transitional Cell) OR(Cell Carcinoma, Transitional) OR(Cell Carcinomas, Transitional) OR(Transitional Cell Carcinoma) OR(Transitional Cell Carcinomas) OR carcinoma of renal calyx[Title/Abstract] OR renal pelvic carcinoma[Title/Abstract] OR renal pelvis cancer[Title/Abstract] OR carcinoma of renal pelvis[Title/Abstract] OR Upper tract urothelial carcinoma[Title/Abstract] OR UTUC[Title/Abstract] OR renal pelvic[Title/Abstract] OR Malignant tumor of the ureter[Title/Abstract] OR eoplasm, Ureteral[Title/Abstract] OR Ureteral Neoplasm[Title/Abstract] OR Neoplasms, Ureteral[Title/Abstract] OR Ureter Neoplasms[Title/Abstract] OR Ureter Neoplasm[Title/Abstract] OR Neoplasms of Ureter[Title/Abstract] OR Cancer of Ureter[Title/Abstract] OR Ureter Cancers[Title/Abstract] OR Ureter, Cancer Of[Title/Abstract] OR Ureter Cancer[Title/Abstract] OR Ureteral Cancer[Title/Abstract] OR Cancer, Ureteral[Title/Abstract] OR Cancers, Ureteral[Title/Abstract] OR Ureteral Cancers[Title/Abstract] OR Cancer of the Ureter[Title/Abstract] OR Urinary Bladder Neoplasms[Title/Abstract] OR Neoplasm, Urinary Bladder[Title/Abstract] OR Urinary Bladder Neoplasm[Title/Abstract] OR Bladder Tumors[Title/Abstract] OR Bladder Tumor[Title/Abstract] OR Tumor, Bladder[Title/Abstract] OR Tumors, Bladder[Title/Abstract] OR Neoplasms, Bladder[Title/Abstract] OR Bladder Neoplasms[Title/Abstract] OR Bladder Neoplasm[Title/Abstract] OR Neoplasm, Bladder[Title/Abstract] OR Urinary Bladder Cancer[Title/Abstract] OR Cancer, Urinary Bladder[Title/Abstract] OR Malignant Tumor of Urinary Bladder[Title/Abstract] OR Cancer of the Bladder[Title/Abstract] OR Bladder Cancer[Title/Abstract] OR Bladder Cancers[Title/Abstract] OR Cancer, Bladder[Title/Abstract] OR Cancer of Bladder[Title/Abstract] | 102660 |
| 3 | randomized controlled tria[Title/Abstract] OR controlled clinical trial[Title/Abstract] OR randomized[Title/Abstract] OR placebo[Title/Abstract] OR clinical trials as topic[Title/Abstract] OR randomly[Title/Abstract] OR trial[Title/Abstract] | 1532323 |
| 4 | 1 AND 2 AND 3 | 105 |
